# Does Masked Hypertension Cause Early Left Ventricular Impairment in Youth?

**DOI:** 10.3389/fped.2018.00167

**Published:** 2018-06-05

**Authors:** Xiu-Xia Luo, Yongsheng Zhu, Yiqian Sun, Quanrong Ge, Jin Su, Hung-Kwan So, Man-Ching Yam, Fang Fang

**Affiliations:** ^1^Department of Ultrasonography, Shenzhen Hospital, Southern Medical University, Shenzhen, China; ^2^Department of Paediatrics and Adolescent Medicine, The University of Hong Kong, Hong Kong, China; ^3^Department of Pediatrics, Prince of Wales Hospital, The Chinese University of Hong Kong, Hong Kong, China; ^4^Beijing Institute of Heart, Lung and Blood Vessel Diseases, Beijing Anzhen Hospital, Capital Medical University, Beijing, China

**Keywords:** masked hypertension, youth, two-dimensional echocardiography, layer-specific speckle tracking analysis, left ventricular function

## Abstract

**Objectives:** Masked hypertension (MH) is not uncommon in the youth and may increase risks of long-term cardiovascular impairment. However, little is known about the subclinical heart damage in this group of patients. Currently, 3-layer speckle tracking imaging based on two-dimensional echocardiography is feasible to detect the early signs of myocardial damage. We therefore aimed to investigate whether subtle changes of cardiac function occurred in the young MH patients by using advanced quantification with layer-specific speckle tracking.

**Methods:** A total of 40 adolescents with MH (age 18 ± 3 years, 73% males) and 40 age-, gender-, race-, and height-matched normotensive volunteers were enrolled in our study. MH was defined as one or more of the ambulatory blood pressure (BP) parameters (24-h, daytime and night-time average BPs) higher than ≥ 95th percentile for gender and height according to the local reference. Both comprehensive two-dimensional echocardiography with layer-specific strain analysis and 24-h ambulatory BP monitoring were performed. Longitudinal strain and circumferential strain in endocardial, mid-myocardial, and epicardial layers were determined accordingly with the dedicated software (EchoPAC software version 201, GE Healthcare, Horten, Norway).

**Results:** Compared with normotensive controls, youths with MH had higher ambulatory pulse rate and left ventricular mass index, and were more obese. Interestingly, similar ventricular volumes and ejection fraction were observed in the study groups, but further analysis with layer-specific strains revealed that endocardial and mid-myocardial longitudinal and circumferential mechanical function were decreased in the young MH subjects when compared to normotensive individuals (all *p* < 0.05). However, there were no difference regarding radial strain and apical rotation derived from traditional speckle tracking analysis.

**Conclusion:** Subclinical change of LV mechanic function assessed by layer-specific speckle tracking is present in youth with MH despite considered as normal with conventional ways.Thus, MH in youth should be monitored closely instead of labeling as an entirely benign entity.

## Introduction

Masked hypertension (MH) has recently become a topic of interest in youths; it refers to a condition in which office blood pressure (BP) is normal but being elevated during ambulatory BP monitoring (1). Actually, MH is not uncommon in the youth. The prevalence ranges from 7.6% in 592 unselected children to 9.4% in 85 youths referred for hypertension evaluation and to 11.9% in 1445 Chinese children from a large territory-wide school-based cohort in Hong Kong (2–4). A growing body of evidence suggests that MH in adults has been associated with more extensive target organ damage than in true normotensive subjects, and similar to that in people with sustained hypertension (5), such as higher left ventricular mass index and thickening of the carotid wall (2). Hence, it is reasonable to infer that MH in youths may increase risks of long-term cardiovascular impairment. Consequently, comprehensive assessment of myocardial function at an early stage may be of clinical importance in MH patients and might provide more information in the risk stratification of these patients.

Two-dimensional (2D) speckle-tracking echocardiography (STE), capable of obtaining quantitative measurements of whole-layer longitudinal, circumferential, and radial directional deformation without the angle dependency of one-dimensional techniques, has been well recognized to allow easily early detection of myocardial damage while left ventricular (LV) ejection fraction (LVEF) is still preserved in various pathologies (6–9). Recently, technological progression of 2D-STE software has enabled the assessment of layer-specific strain, thus allowing to comprehensively evaluate myocardial deformation layer by layer (endocardial, mid-myocardial and epicardial levels), which seems superior in detecting subtle deteriorations of cardiac contractility. Moreover, a report from the EACVI-ASE Strain Standardization Task Force released the feasibility, accuracy, and reproducibility of layer-specific global longitudinal strain measurement (10). Furthermore, the clinical usefulness of the layer-specific technology has already been successfully tested in some clinical settings, including coronary artery disease, myocardial infarction, arterial hypertension, and heart failure (11–16). Of note, recent analysis revealed the layer-specific impairment of LV mechanical function in adult MH patients (age > 50 years old) (8). However, the influence of MH on LV mechanics at its early stage has not been investigated yet.

Thus, this study for the first time provided the new insight to LV mechanical function in youths with MH, and assessed the occurrence of early echocardiographic signs of subclinical left ventricular dysfunction in this young group, using advanced quantification with layer-specific 2D speckle tracking.

## Materials and methods

### Study population

This was a community-based cohort study, which initially enrolled 1445 ethnic Chinese students for surveying 24-h ambulatory BP in Hong Kong during 2011/2012. The presented local reference was used for the definition of MH in Chinese adolescents as one or more of the ambulatory BP parameters (24-h, daytime and night-time average BPs) were higher than 95th percentile for age, gender and height, and the office BPs were normal (4, 17). Totally, 165 subjects (11.9%) were identified as MH at baseline. From June 2015 to July 2016, subjects diagnosed with MH at baseline were invited to recheck ambulatory BP in our center. However, fourteen subjects refused to consent and 7 subjects were lost contact. Among the remaining 144 cases, only 40 (aged from 13 to 22 years) were confirmed with persistent MH and performed echocardiography in our study. Accordingly, 40 normotensive controls, matched with cases for sex, age, race, and body height, were randomly selected from the original cohort. The office BP, 24-h, daytime and night-time average systolic blood pressure (SBP) and diastolic blood pressure (DBP) in controls were all below the sex-specific, age-specific and height-specific 95th percentile during the latest survey. Exclusion criteria were as follows: (1) concomitant pathologies known to impair myocardial contraction (e.g., diabetes, myocardial infarction, and hypertension); (2) uncontrolled arrhythmia; (3) other severe comorbidities that may affect cardiac function; (4) poor echocardiographic image quality or (5) patient unwillingness.

Anthropometric measurements (height, weight) and laboratory analyses (level of plasma fasting glucose, cholesterol and triglycerides) were obtained from all the subjects included in the study. Body mass index (BMI) and body surface area (BSA) were calculated for each subject. The study was approved by the Joint the Chinese University of Hong Kong and New Territories East Cluster Clinical Research Ethics Committee and the Ethics Committee of the Department of Health of the Hong Kong Government (CRE-2013.563). Informed consent was obtained from the participants and their parents before commencement of the monitoring.

### Measurement of office BP

Conventional BP measurements were obtained by measuring the average value of the 3 consecutive measurements in the sitting position, at least 1 min apart and after 5 min of rest, using the Datascope Accutorr Plus (Datascope Corporation, Mahwah, New Jersey, USA), which has been validated in our local Chinese children and adolescents (17, 18). Appropriate cuff size was used according to bladder width (at least 40% of the arm circumference) and length (6 × 12, 9 × 18, or 10 × 24 cm to cover 80–100% of the individual's arm circumference) (19). Two resting office BP measurements were taken before mounting and after dismounting the 24-h BP monitoring device on the following day. A third measurement was taken within 4 weeks for those who had both readings high. The reference values for defining high BP in the office are based on our previous local data (17).

### 24-h ambulatory BP monitoring

All the participants underwent a 24-h BP monitoring. The non-invasive 24-h ambulatory BP monitoring was performed on the non-dominant arm during a weekday with typical activities, using a portable non-invasive oscillometric recorder (TM-2430, A&D Company Limited, Tokyo, Japan). The ABP readings will be obtained every 30 min over a 24-h period. Only ABP profiles with at least 40 recordings, including at least eight readings during sleep, will be accepted. Individual mean systolic, diastolic and mean arterial BP will be calculated for wake and sleep periods. All readings taken during 24 hours will be used to calculated mean 24-h, mean daytime and mean night-time SBP and DBP, in accordance with the method proposed by Soergel et al. (20). Local reference will be used for the definition of ambulatory hypertension as 24-h, daytime or night-time ambulatory BP higher than 95th percentile for gender and height (17). Subjects with office normotension and ambulatory hypertension will be characterized as MH.

### Echocardiography

Echocardiographic examination was performed using a Vivid E9 system (GE Healthcare, Horten, Norway), within 2–3 days after 24-h BP monitoring. Routine 2D cine loops of three consecutive cardiac cycles were obtained at end-expiratory apnea from parasternal long-axis and short-axis views and the standard three apical views (four-chamber, two-chamber, and long-axis) by the same operator. Grayscale recordings were optimized for LV evaluation at a frame rate between 50 and 70 fbp and saved for subsequent strain analyses. LV volume and LVEF were calculated with biplane Simpson method. Left ventricular mass index was determined according to the corrected method of the American Society of Echocardiography (ASE) and indexed to body height^2.7^ (2). The peak early (E) and late (A) transmitral flow velocities during diastole were obtained by pulsed-wave Doppler echocardiography, with the sample volume placed at the mitral leaflet tips, and then the ratio of early-to-late peak velocities (E/A) was calculated. Early diastolic septal mitral annular velocities (e′) were obtained from pulse wave velocity of spectral tissue Doppler imaging and the E/e′ ratio was then calculated (21).

### Two-dimensional LV strain analysis

**Figure 1:**
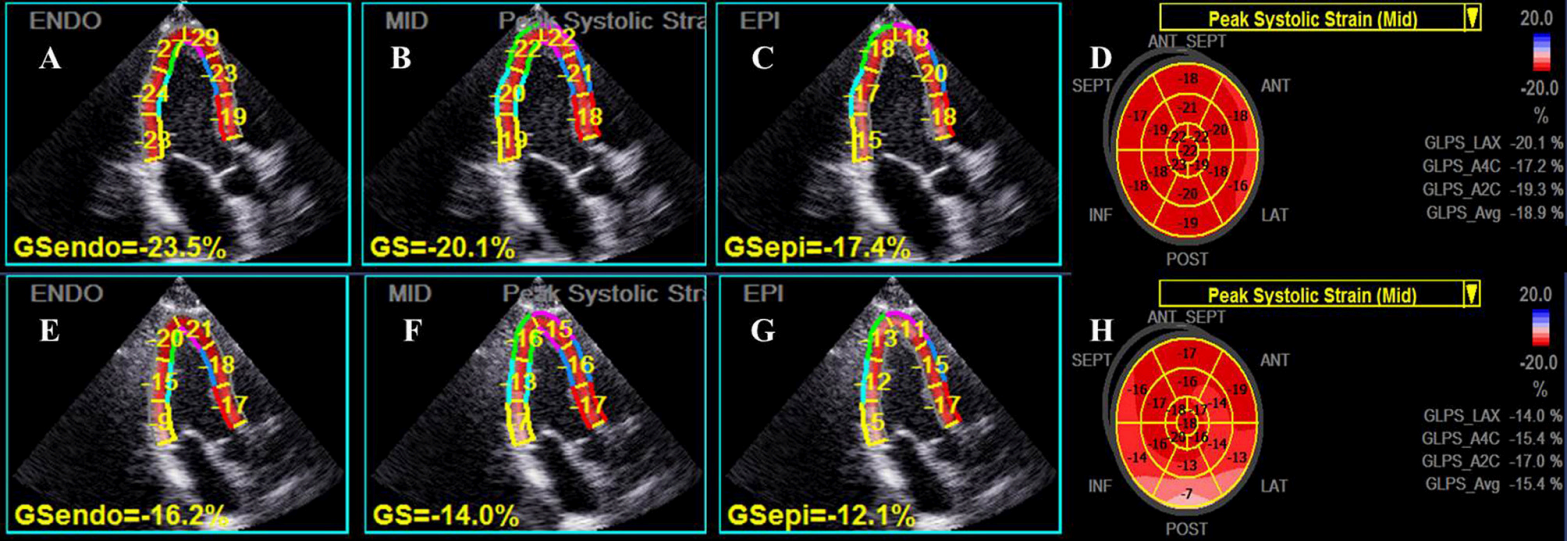
Representative layer-specific longitudinal strain (LS) analyses in apical three-chamber view. The upper row, from left to right panel **(A–D)**, showed the endocardial, mid-myocardial and epicardial layer as well as global mid-myocardial LS results respectively in an individual from the control group, while the lower row **(E–H)** showed the corresponding LS values in a young patient with masked hypertension. Significantly reduced values of LS for all myocardial layers were presented in the patient.

All data of 2D speckle tracking were analyzed off-line using the dedicated EchoPAC V201 software (GE Healthcare, Horten, Norway). The advanced software automatically tracked the contour of endocardium to cover the myocardial thickness of the entire LV wall at an end-diastolic frame, similarly to traditional 2D-STE method, but allowing investigation of three myocardial layers for longitudinal and circumferential strain: endocardial, mid-myocardial and epicardial level. Adequate tracking could be verified in real time and corrected by manual to ensure optimal tracking of endocardium and epicardium and inclusion of the entire LV thickness including endocardial, mid-myocardial and epicardial layers in all observed echocardiographic views (Figure 1). Layer-specific longitudinal strain (LS) was analyzed from the three standard apical views, whereas layer-specific circumferential strain (CS) and radial strain (RS) were evaluated in short axis view at the papillary muscles level. Global strain was calculated as the average of all three layer-specific strains (endocardial, mid-myocardial, and epicardial). The global RS was the mean value of 6 segments from papillary muscles level. The LV apical rotation was measured from parasternal apical level.

### Reproducibility analysis

Intra-observer and inter-observer variabilities of 2D specific-layer strain data were evaluated in 15 randomly subjects by two sonographers blindly. Intra- and inter-observer variabilities were evaluated by means of intraclass correlation coefficients (ICCs).

### Statistical analysis

Collected data were computerized and analyzed using the IBM SPSS Statistics, version 20 (IBM) software. Continuous variables are expressed as mean ± SD and nominal variables as percentages. Data were compared between 2 groups using the independent sample *t*-tests for continuous variables, as well as the chi-square test and Fisher's exact test for categorical variables. The *P* < 0.05 was considered statistically significant.

## Results

### Subject characteristics

None of subjects was found to be primary or secondary hypertensive. Patient characteristics are presented in Table 1. The study groups were of similar age, gender and height distribution. However, BSA and BMI were higher in young MH patients than in controls. Compared with normotensive subjects, participants with MH had higher office BPs as well as ambulatory pulse rates over 24 hours, daytime, and night-time. Also, young MH subjects had higher plasma glucose and triglycerides level than normal controls. There was no difference in heart rate and cholesterol levels between the both groups.

**Table 1:** Clinical characteristics.

**Variables**	**Controls (*n* = 40)**	**MH (*n* = 40)**	***P*-value**
Ages, years	18.3 ± 2.9	18.0 ± 2.6	0.656
Male, *n* (%)	29 (73)	29 (73)	1.000
Height, cm	168 ± 9	167 ± 7	0.784
Weight, kg	58 ± 12	67 ± 15	0.006
BSA, m^2^	1.65 ± 0.20	1.75 ± 0.19	0.026
BMI, kg/m^2^	20 ± 3	24 ± 5	0.001
Heart rate, bpm	64 ± 9	67 ± 10	0.136
Office systolic BP, mm Hg	112 ± 9	118 ± 9	0.002
Office diastolic BP, mm Hg	64 ± 7	69 ± 7	0.002
24-h systolic BP, mm Hg	107 ± 7	123 ± 6	<0.001
24-h diastolic BP, mm Hg	62 ± 5	71 ± 4	<0.001
Daytime systolic BP, mm Hg	112 ± 7	127 ± 8	<0.001
Daytime diastolic BP, mm Hg	66 ± 6	75 ± 5	<0.001
Night-time systolic BP, mm Hg	100 ± 7	114 ± 9	<0.001
Night-time diastolic BP, mm Hg	56 ± 4	64 ± 5	<0.001
Fasting Plasma Glucose, mmol/L	4.7 ± 0.3	5.0 ± 0.4	0.006
Triglycerides, mmol/L	0.7 ± 0.3	0.9 ± 0.5	0.049
Total cholesterol, mmol/L	4.0 ± 0.7	4.1 ± 0.7	0.878
LDL cholesterol, mmol/L	2.2 ± 0.6	2.4 ± 0.6	0.143
HDL cholesterol, mmol/L	1.5 ± 0.3	1.2 ± 0.5	0.005

*Data are presented as mean ± SD or percentages*.

*BSA, body surface area; BMI, body mass index; BP, blood pressure; MH, masked hypertension*.

### Conventional echocardiographic measurements

Routine echocardiographic features of the study population were shown in Table 2. Left ventricular mass indexed to body height^2.7^ was significantly higher in cases than in normotensive individuals. There was no significant difference in the LV wall thickness, ventricular volumes and LVEF between the observed groups. Interestingly, young subjects with MH had lower mitral E/A ratio and mitral annulus e′ when compared with normotensive individuals (all *P* < 0.05), but no difference in E/e′.

**Table 2:** Left ventricular geometry and cardiovascular function.

**Variables**	**Controls (*n* = 40)**	**MH (*n* = 40)**	***P*-value**
LVEDD, mm	46 ± 5	48 ± 4	0.022
LVESD, mm	31 ± 4	32 ± 3	0.272
IVS, mm	7.3 ± 1.3	7.6 ± 1.5	0.214
PWT, mm	7.2 ± 1.1	7.0 ± 1.3	0.853
RWT	0.32 ± 0.05	0.31 ± 0.05	0.506
LVM, g	104 ± 30	115 ± 33	0.111
LVM/height^2.7^, g/m^2.7^	25.4 ± 5.8	28.6 ± 6.5	0.025
LVEDV, mL	101 ± 22	99 ± 17	0.618
LVSV, mL	62 ± 12	64 ± 11	0.399
LVEF, %	61 ± 5	63 ± 4	0.113
E/A ratio	2.5 ± 0.7	2.2 ± 0.6	0.054
Mitral annulus e′, cm/s	13.7 ± 1.7	12.3 ± 1.6	<0.001
E/e′	6.7 ± 1.4	7.2 ± 1.7	0.133

*Cases and controls were matched for sex, age, race and body height*.

*MH, masked hypertension; LVEDD, left ventricular end-diastolic dimension; LVESD, left ventricular end-systolic dimension; IVS, interventricular septum; PWT, posterior wall thickness; RWT, relative wall thickness; LVM, left ventricular mass; LVEDV, left ventricular end-diastolic volume; LVSV, left ventricular stroke volume; LVEF, left ventricular ejection fraction; E, early diastolic mitral flow (pulsed Doppler); A, late diastolic mitral flow (pulse Doppler); e′, peak early diastolic relaxation velocity of the septal mitral annulus (tissue Doppler)*.

### 2D strain measurements

2D-STE derived LV global LS and CS were significantly lower in youth with MH than in controls. Moreover, comparing with true normotensives, LV systolic and early diastolic longitudinal strain rates in MH subjects were significantly decreased, while late diastolic strain rates tended to increase in MH, although without statistically significance (Table 3). However, radial and rotational mechanics was mostly similar between the observed groups.

**Table 3:** Comparison of myocardial strain parameters for study groups.

**Variables**	**Controls (*n* = 40)**	**MH (*n* = 40)**	***P*-value**
**TWO-DIMENSIONAL MECHANICAL PARAMETERS**			
GLS, %	−18.9 ± 1.7	−18.0 ± 1.8	0.026
GCS, %	−20.9 ± 2.6	−19.2 ± 2.2	0.002
GRS, %	49.0 ± 13.1	51.2 ± 13.4	0.468
Apical rotation, degree	13.6 ± 4.9	13.5 ± 5.8	0.935
Systolic longitudinal strain rate, s^−1^	−1.03 ± 0.1	−0.97 ± 0.1	0.019
Early longitudinal diastolic strain rate, s^−1^	1.84 ± 0.2	1.66 ± 0.3	0.001
Late longitudinal diastolic strain rate, s^−1^	0.50 ± 0.1	0.54 ± 0.1	0.098
**TWO-DIMENSIONAL LAYER-SPECIFIC STRAINS**			
Endocardium GLS, %	−21.6 ± 1.9	−20.4 ± 1.9	0.007
Mid-myocardium GLS, %	−18.8 ± 1.7	−17.8 ± 1.7	0.017
Epicardium GLS, %	−16.3 ± 1.5	−15.8 ± 1.9	0.164
Endocardium GCS, %	−30.4 ± 3.4	−28.9 ± 3.0	0.037
Mid-myocardium GCS, %	−19.8 ± 2.5	−18.0 ± 2.2	0.001
Epicardium GCS, %	−12.5 ± 2.1	−10.6 ± 1.9	<0.001

*GLS, global longitudinal strain; GCS, global circumferential strain; GRS, Global radial strain*.

**Figure 2:**
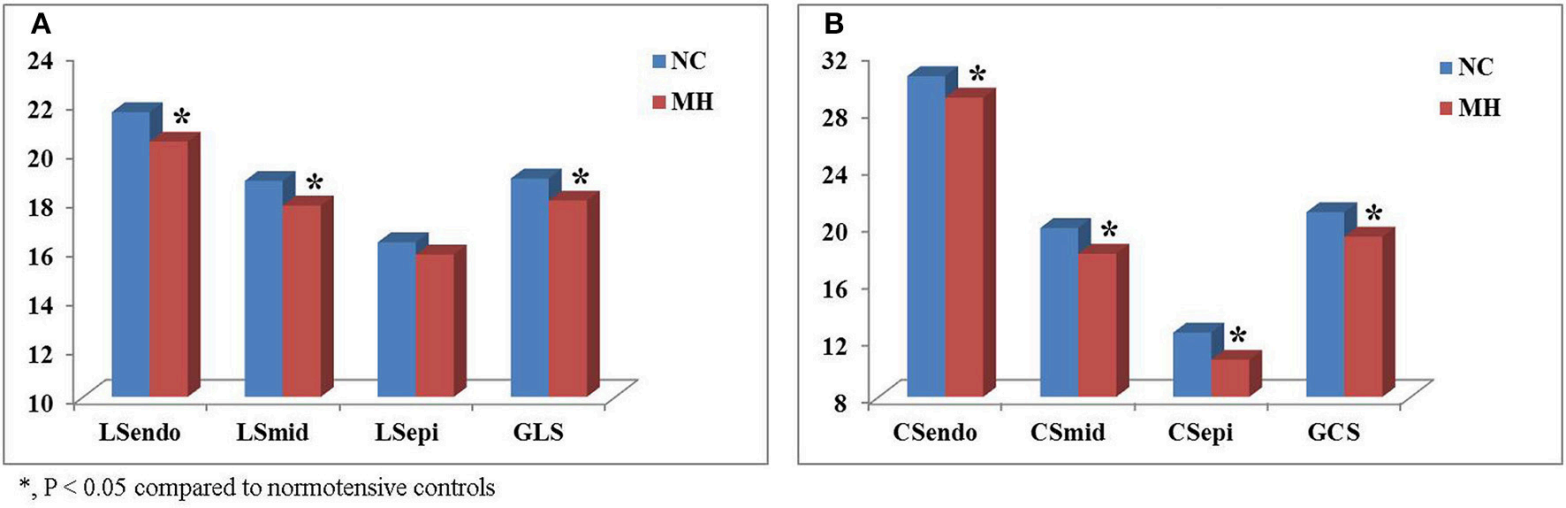
Comparison of layer-specific strains between young subjects with masked hypertension (MH) and normotensive controls (NC). Left ventricular global longitudinal (LS) **(A)** and circumferential strain (CS) (B) were significantly lower in youth with MH than in normotensive controls. Moreover, layer-specific strain analysis showed that endocardial and mid-myocardial LS **(A**) and CS **(B)** during systole were significantly worsened in the young MH individuals when compared to controls. LSendo, endocardial longitudinal strain; LSmid, mid-myocardial longitudinal strain; LSepi, epicardial longitudinal strain; GLS, global longitudinal strain; CSendo, endocardial circumferential strain; CSmid, mid-myocardial circumferential strain; CSepi, epicardial circumferential strain; GCS, global circumferential strain. *P < 0.05.

Further layer-specific 2D strain analysis revealed that endocardial and mid-myocardial longitudinal and circumferential mechanical functions during systole were significantly worsened in the young MH individuals when compared to normotensive controls (all *p* < 0.05) (Table 3 and Figure 2).

### Intra- and inter-observer variability

As it has been shown in Table 4, intra- and inter-observer agreement are high for all parameters of 2D-STE LV multilayer strains.

**Table 4:** Intra- and inter-observer variability of layer-specific strain parameters.

	**Longitudinal strain**			**Circumferential strain**		
	**ICCs**	**95% CI**	**P**	**ICCs**	**95% CI**	**P**
**INTRA-OBSERVER VARIABILITY**						
Endocardium	0.972	0.917–0.991	<0.001	0.888	0.665–0.962	<0.001
Mid-myocardium	0.984	0.954–0.995	<0.001	0.839	0.520–0.946	0.001
Epicardium	0.993	0.978–0.998	<0.001	0.835	0.509–0.945	0.001
**INTER-OBSERVER VARIABILITY**						
Endocardium	0.940	0.824–0.980	<0.001	0.801	0.418–0.933	0.003
Mid-myocardium	0.951	0.818–0.985	<0.001	0.845	0.542–0.948	0.001
Epicardium	0.950	0.757–0.986	<0.001	0.896	0.691–0.965	<0.001

*ICCs, intraclass correlation coefficients; CI, confidence interval*.

## Discussion

Recent studies have demonstrated that the prevalence of MH in youth is more common than previously reported and appears to be more closely associated with increased cardiovascular risk in later adulthood (2–5). Consequently detection of subtle LV contractile dysfunction at an early subclinical stage allows earlier intervention to avoid irreversible consequent LV deterioration. Of note, 2D-STE is a rapidly evolving tool for the assessment of myocardial deformation in children (22), without the angle dependency of one-dimensional technique. Moreover, the advanced strain analysis has been supported by the latest guidelines that recommended determination of LV strains in adult hypertension, due to its high predictive value that is even higher than for conventional LVEF (23). Recent clinical studies had reported that LV myocardial strain is a sensitive marker of early cardiac dysfunction, and is associated with outcomes in pediatric disease processes known to impair contractility, such as diabetes mellitus, chemotherapy cardiotoxicity, amyloidosis, Kawasaki disease, etc. (6, 24–26). Nevertheless, no data has provided any information on the MH in the youth.

In the present study, we have compared measurements of cardiac function between hypertensive and normotensive children. Similar to the previous studies, patients with MH had higher ambulatory pulse rate and plasma glucose level than normotensive subjects, and were more obese (3, 27). However, commonly used echocardiographic parameters of systolic function including LV volumes and LVEF were not significantly different between the study groups. Therefore, our study for the first time provided insight in LV structural, functional, and mechanical changes in young MH patients by using the advanced and more sensitive layer-specific strain analysis.

Importantly, our results revealed that the young MH subjects were associated with subclinical LV systolic dysfunction, which can be identified as a reduction in longitudinal and circumferential LV deformations and, particularly, in endocardial and mid-myocardial layers. Thus, our study confirms the application of these measurements for early detection of myocardial impairment consistent with recently published comparable results in adult MH patients (8), which demonstrated significantly reduced LS and CS of all three LV myocardial layers (endocardial, mid-myocardial and epicardial) in adult masked hypertensives.

It is still controversial regarding the number of layers in myocardial wall. Nevertheless, there is the agreement that the myocyte arrangement in different layers is different (8, 13). Endocardial and epicardial fibers are mainly longitudinally oriented, whereas the mid-myocardial fibers are circumferentially directed (8, 20). Moreover, previous study revealed a decreasing gradient layer-specific strain from the endocardium to the epicardium in both controls and adult patients with hypertension (13–28). Using animal model, investigators found that the endocardium was most vulnerable to the effect of LV filling pressure, and its function was easily impaired in patients with hypertension (29). Furthermore, considering that LV hypertrophy occurs from endocardium to epicardium, with myocytes hypertrophy, fibroblast proliferation and interstitial changes (29–31), it is essential to assess the influence of hypertrophy on each myocardial layer. Interestingly, our findings showed that, LS and CS of endocardial and mid-myocardial layers have been affected in young MH subjects, which indicated that deterioration of LV mechanical function was spreading from endocardium to epicardium even in subjects who do not have sustained BP elevation.

On the other hand, we observed that LV apical rotation was preserved in the MH group. This is similar with the previous hypertension study in pediatrics, which demonstrated no statistical difference of LV rotation in children and adolescents with systemic arterial hypertension compared with those with normotension (32). A potential explanation could be that in the young cohort with MH or hypertension, the disease state had not been presented for long enough to induce an impact on the myocardium to impair LV apical rotation.

As for LV diastolic function, the conventional echocardiographic parameters like E/A ratio and mitral annulus e′, were decreased in the MH group, when compared to the normal controls. Also, the E/e′ ratio, a surrogate for filling pressure, tended to be higher in masked hypertensions despite no statistically difference between the observed groups. Of note, we for the first time applied the advanced speckle-tacking analysis to evaluate LV diastolic function in subjects with MH. In line with the previous studies in adult MH patients, our findings showed that LV longitudinal strain rates during systole and early diastole were significantly lower in this young MH group than in confirmed normotensive subjects, whereas the late diastolic strain rate tended to increase in MH, although without statistically significance.

Previous studies have demonstrated MH is closely associated with obesity and target-organ damage in adolescents (33–35). Moreover, obesity and higher plasma glucose level in youth has been found to be linked to decreased myocardial deformation even in the absence of comorbidities in early stages (24, 36–38). Therefore, it is reasonable to indicate that the observation of reduction of strain in asymptomatic young MH patients may contribute to the comprehensive effects of higher ambulatory pulse rate and glucose level as well as body weight. The studies regarding the influence of LV mechanics on cardiovascular outcome in youths with MH have not been done so far. However, considering our results that revealed the decrements of both longitudinal and circumferential layer-specific strain and abnormal diastolic LV filling in asymptomatic young MH subjects, and the findings of recent longitudinal study regarding the predictive value of myocardial strain (39), it could be hypothesized that LV mechanical changes might contribute to higher morbidity and mortality of MH patients later in life. Hence, MH in youths should be regarded as a condition that requires periodic follow-up.

The main limitation in the present study is lack of a “gold standard” like cardiac magnetic resonance imaging (MRI) to validate the layer-specific strain determined by 2D-STE. However, Altiok E et al. reported that the layer-specific strain using cardiac MRI demonstrated good correlations with the layer-specific strain using 2D echocardiography (40). Of note, a report from the EACVI-ASE Strain Standardization Task Force has also released the feasibility, accuracy, and reproducibility of layer-specific global longitudinal strain measurement (10). Second, 2D-STE assessment of LV mechanics could be influenced by the quality of ultrasound images. Manual drawing of the cardiac border also needs special care. Also, 2D-STE analysis was performed on a single analysis platform, so our results may not be able to be extrapolated to different analysis software. Third, the reference values for defining high BP in the office are based on our previous local data, instead of established BP normative data for children (including CDC and WHO data). However, it was contributed to the age of studied subjects were ranged from 13 to 22 years, and using different references of the ambulatory blood pressure might reduce or increase the number of hypertensive patients. Moreover, the BP normative data from CDC or WHO did not include the data from the Chinese, while the ethnic difference might cause misleading in the interpretation. Finally, the investigation only covered a relatively small number of young subjects with MH in a single-center study and a larger study is required to further confirm our results. Nevertheless, major strengths of our study are the nature of school-based population, instead of hospital-based cohorts.

## Conclusions

With layer-specific 2D-STE, subclinical impairment of LV mechanical function in different myocardial layers is present in youth with MH, despite considered as normal with conventional ways. Thus, periodic follow-up and early detection of LV functional deterioration in young MH patients may help to prevent the natural progression of the disease.

## Author contributions

M-CY, H-KS, and FF conceived and designed the experiments. X-XL, H-KS, and YZ organized the database. X-XL and YS performed the echocardiographic images analysis and statistical analysis. X-XL wrote the first draft of the manuscript. X-XL, YS, QG, and JS wrote sections of the manuscript. All authors have read and revised the manuscript, which was coordinated by FF and YZ, who also produced the final draft for submission.

### Conflict of interest statement

The authors declare that the research was conducted in the absence of any commercial or financial relationships that could be construed as a potential conflict of interest.

## Abbreviations

MH: masked hypertension
BP: blood pressure
2D: two-dimensional.2D-STE, two-dimensional speckle-tracking echocardiography
LV: left ventricle
LVEF: left ventricular ejection fraction
2DE: two-dimensional echocardiography
SBP: systolic blood pressure
DBP: diastolic blood pressure
BMI: body mass index
BSA: body surface area
ASE: the American Society of Echocardiography
E: peak early transmitral flow velocities during diastole
A: peaklatetransmitral flow velocities during diastole
e′: early diastolic septal mitral annular velocities
LS: longitudinal strain
CS: circumferential strain
RS: radial strain
ICCs: intraclass correlation coefficients
MRI: magnetic resonance imaging.

## References

[1] Pickering TG Davidson K Gerin W Schwartz JE. Masked hypertension. Hypertension (2002) 40:795–6. 10.1161/01.HYP.0000038733.08436.98 12468559

[2] Stabouli S Kotsis V Toumanidis S Papamichael C Constantopoulos A Zakopoulos N. White-coat and masked hypertension in children: association with target-organ damage. Pediatr Nephrol. (2005) 20:1151–5. 10.1007/s00467-005-1979-5 15947982

[3] Lurbe E Torro I Alvarez V Nawrot T Paya R Redon J et al. Prevalence, persistence, and clinical significance of masked hypertension in youth. Hypertension (2005) 45:493–8. 10.1161/01.HYP.0000160320.39303.ab 15767467

[4] So HK Yip GW Choi KC Li AM Leung LC Wong SN et al. Association between waist circumference and childhood-masked hypertension: a community-based study. J Paediatr Child Health (2016) 52:385–90. 10.1111/jpc.13121 27145500

[5] Verberk WJ Kessels AG de Leeuw PW. Prevalence, causes, and consequences of masked hypertension: a meta-analysis. Am J Hypertens. (2008) 21:969–75. 10.1038/ajh.2008.221 18583985

[6] Buss SJ Emami M Mereles D Korosoglou G Kristen AV Voss A et al. Longitudinal left ventricular function for prediction of survival in systemic light-chain amyloidosis: incremental value compared with clinical and biochemical markers. J Am Coll Cardiol. (2012) 60:1067–76. 10.1016/j.jacc.2012.04.043 22883634

[7] Luo XX Fang F Yu CM. Advantageous effect of biventricular pacing on cardiac function and coronary flow: a case report. Int J Cardiol. (2015) 190:236–8. 10.1016/j.ijcard.2015.04.151 25930145

[8] Tadic M Cuspidi C Vukomanovic V Celic V Tasic I Stevanovic A et al. Does masked hypertension impact left ventricular deformation? J Am Soc Hypertens. (2016) 10:694–701. 10.1016/j.jash.2016.06.032 27461398

[9] Luo XX Fang F Lee AP Sun JP Li S Zhang ZH et al. What can three-dimensional speckle-tracking echocardiography contribute to evaluate global left ventricular systolic performance in patients with heart failure? Int J Cardiol. (2014) 172:132–7. 10.1016/j.ijcard.2013.12.314 24485606

[10] Ünlü S Mirea O Duchenne J Pagourelias ED Bézy S Thomas JD et al. Comparison of feasibility, accuracy, and reproducibility of layer-specific global longitudinal strain measurements among five different vendors: a report from the EACVI-ASE strain standardization task force. J Am Soc Echocardiogr. (2018) 31:374–80.e1. 10.1016/j.echo.2017.11.008 29246512

[11] Sarvari SI Haugaa KH Zahid W Bendz B Aakhus S Aaberge L et al. Layer-specific quantification of myocardial deformation by strain echocardiography may reveal significant CAD in patients with non-ST-segment elevation acute coronary syndrome. JACC Cardiovasc Imaging (2013) 6:535–44. 10.1016/j.jcmg.2013.01.009 23582354

[12] Tarascio M Leo LA Klersy C Murzilli R Moccetti T Faletra FF. Speckle-tracking layer-specific analysis of myocardial deformation and evaluation of scar transmurality in Chronic Ischemic Heart disease. J Am Soc Echocardiogr. (2017) 30:667–75. 10.1016/j.echo.2017.03.015. 28511861

[13] Kim SA Park SM Kim MN Shim WJ. Assessment of left ventricular function by layer-specific strain and its relationship to structural remodelling in patients with hypertension. Can J Cardiol. (2016) 32:211–6. 10.1016/j.cjca.2015.04.025 26255215

[14] Alcidi GM Esposito R Evola V Santoro C Lembo M Sorrentino R et al. Normal reference values of multilayer longitudinal strain according to age decades in a healthy population: a single-centre experience. Eur Heart J Cardiovasc Imaging (2017). [Epub ahead of print]. 10.1093/ehjci/jex306 29211878

[15] Zhang L Wu WC Ma H Wang H. Usefulness of layer-specific strain for identifying complex CAD and predicting the severity of coronary lesions in patients with non-ST-segment elevation acute coronary syndrome: compared with syntax score. Int J Cardiol. (2016) 223:1045–52. 10.1016/j.ijcard.2016.08.277 27592047

[16] Nishi T Funabashi N Ozawa K Takahara M Fujimoto Y Kamata T et al. Resting multilayer 2D speckle-tracking transthoracic echocardiography for the detection of clinically stable myocardial ischemic segments confirmed by invasive fractional flow reserve. Part 1: vessel-by-vessel analysis. Int J Cardiol. (2016) 218:324–32. 10.1016/j.ijcard.2016.05.016 27259166

[17] Yip GW Li AM So HK Choi KC Leung LC Fong NC et al. Oscillometric 24-h ambulatory blood pressure reference values in Hong Kong Chinese children and adolescents. J Hypertens. (2014) 32:606–19. 10.1097/HJH.0000000000000062 24445392

[18] Wong SN Tz Sung RY Leung LC. Validation of three oscillometric blood pressure devices against auscultatory mercury sphygmomanometer in children. Blood Press Monit. (2006) 11:281–91. 10.1097/01.mbp.0000209082.09623.b4 16932037

[19] Lurbe E Agabiti-Rosei E Cruickshank JK Dominiczak A Erdine S Hirth A et al. 2016 European Society of Hypertension guidelines for the management of high blood pressure in children and adolescents. J Hypertens. (2016) 34:1887–920. 10.1097/HJH.0000000000001039 27467768

[20] Soergel M Kirschstein M Busch C Danne T Gellermann J Holl R et al. Oscillometric twenty-four-hour ambulatory blood pressure values in healthy children and adolescents: a multicenter trial including 1141 subjects. J Pediatr. (1997) 130:178–84. 9042117 10.1016/s0022-3476(97)70340-8

[21] Quiñones MA Otto CM Stoddard M Waggoner A Zoghbi WA. Doppler quantification task force of the nomenclature and standards committee of the american society of echocardiography. recommendations for quantification of doppler echocardiography: a report from the doppler quantification task force of the Nomenclature and Standards Committee of the American Society of Echocardiography. J Am Soc Echocardiogr. (2002) 15:167–84. 11836492 10.1067/mje.2002.120202

[22] Friedberg MK Mertens L. Tissue velocities, strain, and strain rate for echocardiographic assessment of ventricular function in congenital heart disease. Eur J Echocardiogr. (2009) 10:585–93. 10.1093/ejechocard/jep045 19401299

[23] Marwick TH Gillebert TC Aurigemma G Chirinos J Derumeaux G Galderisi M et al. Recommendations on the use of echocardiography in adult hypertension: a report from the European Association of Cardiovascular Imaging (EACVI) and the American Society of Echocardiography (ASE). Eur Heart J Cardiovasc Imaging (2015) 16:577–605. 10.1093/ehjci/jev076 25995329

[24] Yoldaş T Örün UA Sagsak E Aycan Z Kaya Ö Özgür S et al. Subclinical left ventricular systolic and diastolic dysfunction in type 1 diabetic children and adolescents with good metabolic control. Echocardiography (2018). 35:227–33. 10.1111/echo.13764 29205484

[25] Armstrong GT Joshi VM Ness KK Marwick TH Zhang N Srivastava D et al. Comprehensive echocardiographic detection of treatment-related cardiac dysfunction in adult survivors of childhood cancer: results from the St. jude lifetime cohort study. J Am Coll Cardiol.(2015) 65:2511–22. 10.1016/j.jacc.2015.04.013 26065990 PMC4539123

[26] Azak E Cetin II Gursu HA Kibar AE Surucu M Orgun A et al. Recovery of myocardial mechanics in Kawasaki disease demonstrated by speckle tracking and tissue Doppler methods. Echocardiography (2018) 35:380–7. 10.1111/echo.13773 29239028

[27] Björklund K Lind L Zethelius B Andrén B Lithell H. Isolated ambulatory hypertension predicts cardiovascular morbidity in elderly men. Circulation (2003) 107:1297–302. 10.1161/01.CIR.0000054622.45012.12 12628951

[28] Sun JP Xu T Yang Y Yang XS Shang Q Li Y et al. Layer-specific quantification of myocardial deformation may disclose the subclinical systolic dysfunction and the mechanism of preserved ejection fraction in patients with hypertension. Int J Cardiol. (2016) 219:172–6. 10.1016/j.ijcard.2016.06.035 27327503

[29] Nakamura N Hirata K Imanishi T Kuroi A Arita Y Ikejima H et al. Electrocardiographic strain and endomyocardial radial strain in hypertensive patients. Int J Cardiol. (2011) 150:319–24. 10.1016/j.ijcard.2010.04.049 20510470

[30] Tadic M Cuspidi C Ivanovic B Ilic I Celic V Kocijancic V. Influence of white-coat hypertension on left ventricular deformation 2- and 3-dimensional speckle tracking study. Hypertension (2016) 67:592–6. 10.1161/HYPERTENSIONAHA.115.06822 26729750

[31] Tadic M Cuspidi C Vukomanovic V Celic V Pavlovic T Kocijancic V. The influence of masked hypertension on the right ventricle: is everything really masked? J Am Soc Hypertens. (2016) 10:318–24. 10.1016/j.jash.2016.02.001 26948961

[32] Navarini S Bellsham-Revell H Chubb H Gu H Sinha MD Simpson JM. Myocardial deformation measured by 3-dimensional speckle tracking in children and adolescents with systemic arterial hypertension. Hypertension (2017) 70:1142–7. 10.1161/HYPERTENSIONAHA.117.09574 29084877

[33] Lurbe E Invitti C Torro I Maronati A Aguilar F Sartorio A et al. The impact of the degree of obesity on the discrepancies between office and ambulatory blood pressure values in youth. J Hypertens. (2006) 24:1557–64. 10.1097/01.hjh.0000239291.32883.e3 16877958

[34] Stabouli S Kotsis V Papamichael C Constantopoulos A Zakopoulos N. Adolescent obesity is associated with high ambulatory blood pressure and increased carotid intimal-medial thickness. J Pediatr. (2005) 147:651–56. 10.1016/j.jpeds.2005.06.008 16291358

[35] Li AQ Zhao ZY Zhang LL Lu FH Yan ZH Li YY et al. Overweight influence on circadian variations of ambulatory blood pressure in Chinese adolescents. Clin Exp Hypertens. (2005) 27:195–201. 10.1081/CEH-48777 15835382

[36] Kulkarni A Gulesserian T Lorenzo JMMD Haroonian Y Ngyuyen M Lo Y et al. Left ventricular remodelling and vascular adaptive changes in adolescents with obesity. Pediatr Obes. (2018). [Epub ahead of print]. 10.1111/ijpo.12278 29569422

[37] Mangner N Scheuermann K Winzer E Wagner I Hoellriegel R Sandri M et al. Childhood obesity: impact on cardiac geometry and function. JACC Cardiovasc Imaging (2014) 7:1198–205. 10.1016/j.jcmg.2014.08.006. 25306542

[38] Hensel KO Grimmer F Jenke AC Wirth S Heusch A. The influence of real-time blood glucose levels on left ventricular myocardial strain and strain rate in pediatric patients with type 1 diabetes mellitus-a speckle tracking echocardiography study. BMC Cardiovasc Disord. (2015) 15:175. 10.1186/s12872-015-0171-5 26691324 PMC4687137

[39] Lee WH Liu YW Yang LT Tsai WC. Prognostic value of longitudinal strain of subepicardial myocardium in patients with hypertension. J Hypertens. (2016) 34:1195–200. 10.1097/HJH.0000000000000903 27035737

[40] Altiok E Neizel M Tiemann S Krass V Kuhr K Becker M et al. Quantitative analysis of endocardial and epicardial left ventricular myocardial deformation-comparison of strain-encoded cardiac magnetic resonance imaging with two-dimensional speckle-tracking echocardiography. J Am Soc Echocardiogr. (2012) 25:1179–88. 10.1016/j.echo.2012.07.019 22951120

